# Multiple talon cusps on maxillary central incisor: A case report

**DOI:** 10.15171/joddd.2017.023

**Published:** 2017-06-21

**Authors:** Suresh KV, Pramod R C, Seema Roodmal Yadav, Nilesh Kumar, Mounesh Kumar C D, Sreeja P Kumar

**Affiliations:** ^1^Faculty of Dentistry, SEGi University, No. 9 Jalan Teknologi, Taman Sains, Petaling Jaya, Malaysia; ^2^Department of Oral Pathology & Microbiology, Faculty of Dentistry, Batterjee Medical College, Jeddah, Saudi Arabia; ^3^Department of Periodontology, Yogita Dental College & Hospital, Khed (Ratnagiri), Maharashtra, India; ^4^Department of Oral & Maxillofacial Surgery, School of Dental Sciences, Krishna Institute of Medical Sciences, Deemed University, Karad, Satara (District), Maharashtra (State), India.; ^5^Department of Oral Medicine and Radiology, Amrita School of Dentistry, Kochi, Kerala, India

**Keywords:** Dens evaginatus, maxilla, multiple, supernumerary tooth, talon cusp

## Abstract

Dental anomalies affecting the teeth are relatively common. Simultaneous occurrence of multiple dental abnormalities in a single tooth is uncommon and relatively rare. One such abnormality routinely encountered in dental clinics is the talon cusp. It is also referred to as dens evaginatus, characterized by the presence of an accessory cusp-like structure projecting from the cingulum of anterior teeth. It has an increased predilection for maxillary teeth and permanent dentition. Although numerous cases of talon cusp have been reported in the literature, occurrence of multiple talon cusps in maxillary central incisors has not been found in the literature. This case report highlights the presence of talon cusps in maxillary anterior teeth with multiple impacted supernumerary teeth.

## Introduction

Dens evaginatus is a relatively rare developmental anomaly characterized by the presence of an accessory cusp-like structure projecting from the cingulum area or cemento-enamel junction.^1^

Previous researchers have noticed that maxillary lateral incisors have high predilection for developmental anomalies followed by permanent central incisors, canines and molars.^2^ These developmental disturbances occur during the morphogenesis stage. The term 'Talon cusp' was adopted by Mellor and Ripa due to its close resemblance to an eagle's talon. This condition has a predilection for permanent maxillary lateral (55%) and central (33%) incisors, followed by mandibular canines (6%), and maxillary canines (4%).^3^ It has a prevalence rate of 0.06% in Mexican, 7.7% in North Indian, 0.17% in American and 2.5% in Hungarian children.^4^ Talon cusp is composed of normal enamel, dentin and varying extensions of pulp tissue. It can occur alone or in association with dens invaginatus in a few cases.^2^ Clinical management of this developmental anomaly varies from case to case. Normally, it is asymptomatic; however, some patients might have aesthetic and functional problems of occlusal interference and deposition of local factors which may result in the development of caries and periodontitis. The attrition of cusp may expose the pulp horn; hence, prophylactic enameloplasty may be required. Also, desensitizing agent along with regular clinical and radiographic follow-up could be helpful.^5,6^

The occurrence of multiple talon cusps on maxillary central incisors is an extremely rare phenomenon. In addition to it, the present case had talon cusps in maxillary anterior teeth and non-syndromic impacted supernumerary teeth.

## Case report

**Figure 1:**
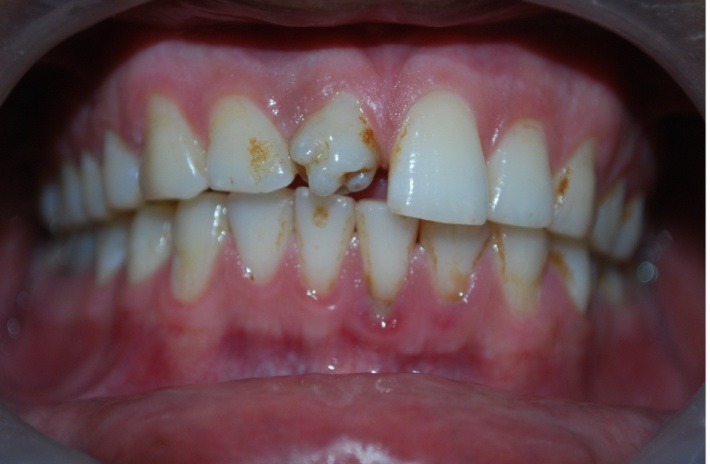
Photograph showing labial and incisor aspect of central incisor.

**Figure 2:**
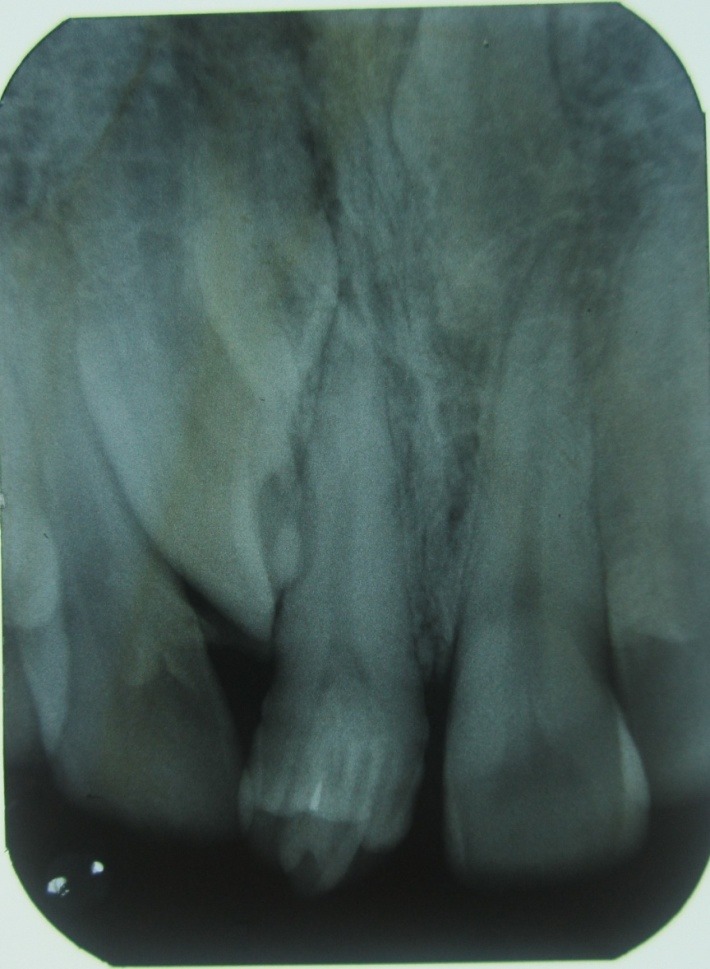
IOPA radiograph showing a central prominent cusp surrounded by small cusps and periapical area showing three impacted supernumerary teeth.

**Figure 3:**
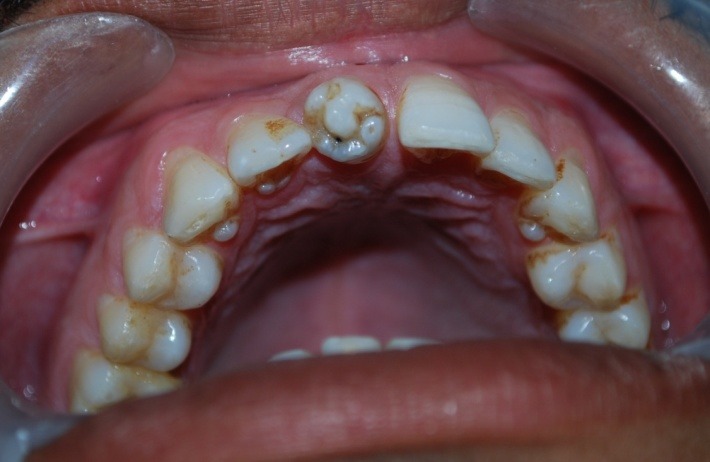
Intraoral photograph showing an unusual and morphologically altered central incisor with 5 small cusps and large central cusp.

A 32-year-old male patient reported to the Department of Oral Medicine and Radiology for routine check-up. This was the patient’s first visit to a dentist. His medical and family histories were non-contributory. There were no findings indicative of any syndrome. An intraoral examination of maxillary right central incisor revealed a morphologically altered crown with multiple small cusps and a prominent central cusp (Figures 1 and 2). There were 5 small cusps and a prominent central cusp. Presence of talon cusp in relation to teeth #12, #13 and #33 was also seen. Mild stains were present on the occlusal surface of central incisor with pit caries. Intraoral periapical radiograph revealed prominent talon cusp at the center surrounded by small cusps along with three impacted supernumerary teeth at the periapex. The root, lamina dura and periodontal ligament space of tooth #11 appeared to be normal (Figure 3). Examination of the remaining dentition showed no obvious abnormalities. The patient was told about the condition. Routine scaling and oral prophylaxis were performed. Prosthetic crown in relation to tooth #11 was planned to correct the esthetic appearance and surgical removal of impacted teeth was suggested. Since the patient was asymptomatic, he refused to undergo extraction of the impacted teeth. However, he was advised to undergo prophylactic enameloplasty of the talon cusps, followed by topical application of a desensitizing agent. He was also informed about the possible consequences of the impacted teeth and a regular clinical and radiographic follow-up of the impacted tooth was suggested.

## Discussion

The exact etiology of talon cusp is unknown; it does appear that both genetic and environmental factors coexist. This condition is believed to occur during morphodifferentiation stage as a result of outward folding of inner enamel epithelial cells and transient focal hyperplasia of dental papilla.^2^ Few authors suggested that an aberrant hyperactivity of the dental lamina and gene mutation at different levels of tooth formation could lead to multiple talon cusps. Morphology of the tooth and the number of cusp formations is controlled by specific genes.^7^ The enamel knots are responsible for determining the shape and number of cusps. They regulate cuspal morphogenesis by the release of various molecules such as fibroblast growth factors 4 and 9, transforming growth factor-β and bone morphogenic proteins 2, 4 and 7. These molecules cause the initiation of secondary enamel knots that mark the cusp formation at early bell stage.^8^

The involved tooth is composed of normal enamel and dentin, either with varying extensions of pulp tissue or without a pulp horn.^2^ Talon cusp can also occur in association with other dental anomalies such as peg-shaped lateral incisors, agenesis of canines, mesiodens, complex odontomas and megadont. Clinical presentation ranges from an enlarged cingulum to a large well-delineated cusp extending beyond the incisal edge of tooth. However, wide variations in the size and shape of this anomaly are seen. Hattab et al classified this anomaly based on the degree of their formation and extension as – talon, semi-talon and trace talon. This was later modified as type 1: major talon, type 2: minor talon and type 3: trace talon.^2,9^

The present case can be classified as type I for the large central cusp and type 2 for the surrounding smaller cusps.

This anomaly is also found to be associated with Rubinstein-Taybi syndrome, Mohr syndrome,  Sturge-Weber syndrome and incontinentia pigmenti.^10^The present case had talon cusps in maxillary anterior teeth with non-syndromic impacted supernumerary teeth. Talon cusp could cause multiple clinical problems, which includes impaired aesthetics, occlusal interference, caries, impaction of teeth, periodontal problems, irritation of the tongue and other diagnostic problems.^11,12^In the present case, the patient had pit caries with brownish stains on the occlusal aspect of the tooth; however, there was no evidence of occlusal interference and periapical pathology. Occurrence of multiple talon cusps in a maxillary central incisor is extremely rare. To the best of our knowledge no such cases have been reported so far. Literature search reveals two cases of multiple talon cusps in supernumerary teeth. Nagaveni et al reported a case of multilobed mesiodens with palatal talon cusp.^13^In this case report, the morphology of the tooth crown was found to be unusual with three lobes separated by non-carious developmental grooves. Dave et al reported a similar type of multi-lobed mesiodens with talon cusp in an 8-year-old patient.^14^ The present case is another contribution to such a rare occurrence of multiple talon cusps in maxillary central incisors with multiple supernumerary teeth to the dental literature. In cases described by Nagaveni et al and Dave et al multi-lobes were seen in supernumerary teeth, but the present case highlights the presence of multiple talon cusps in maxillary central incisor.

Intraoral radiographs play a crucial role in the diagnosis of talon cusps and other associated pathologies. Radiographically, it may appear typically as a V-shaped radiopaque structure, as in true talon or semi-talon, or be tubercle-like, as in trace talon, originating from the cervical third of the root. This appearance varies with the shape and size of the cusp and the angle at which the radiograph is taken.^15^In the present case, the tooth showed a central large projection surrounded by small cusps with no evidence of periapical changes.

The management of talon cusps varies with the clinical presentations of each case and should be as conservative as possible. For deep developmental grooves, simple prophylactic measures such as fissure sealing and composite resin restorations can be carried out. In case of occlusal interference, reduction of the bulk of the cusp gradually and periodically, and application of topical fluoride gel is indicated to reduce sensitivity and to stimulate reparative dentin for pulp protection.^16^

In the present case, multiple cusps resulted in poor esthetics and caries in the grove without any occlusal interference. Prosthetic crown in relation to tooth #11 and extraction of impacted supernumerary teeth were planned. However, the patient refused to undergo any treatment as he was asymptomatic.

## Conclusion

Occurrence of multiple talon cusps in a single tooth is extremely rare and uncommon. These abnormalities can give rise to various clinical complications requiring immediate intervention. It is extremely important for dental practitioners to recognize these anomalies. Early identification and management of these developmental anomalies is mandatory as a part of preventive dentistry.

## Acknowledgments

The authors would like to thank Dr. Akshay Kumarswamy (MS) interim chair division of periodontics, East Carolina University School of Dental Medicine, Greenville NC 27834, USA.

## Authors’ contributions

SKV and PRC were involved in the diagnosis and formatting the case report. SKV and SRY were involved in drafting the manuscript and all the authors contributed to the revision and final approval of the manuscript.

## Funding

The authors report no funding for this article.

## Competing interests

The authors declare no competing interests with regards to the authorship and/or publication of this article.

## Ethics approval and Patient consent

The authors declare that the patient, whose data was reported in this article, has given written consent to the authors and for the publication of this paper.
